# Diversity and occurrence of methylotrophic yeasts used in genetic engineering

**DOI:** 10.18699/VJ20.602

**Published:** 2020-03

**Authors:** A.S. Rozanov, E.G. Pershina, N.V. Bogacheva, V. Shlyakhtun, A.A. Sychev, S.E. Peltek

**Affiliations:** Institute of Cytology and Genetics of Siberian Branch of the Russian Academy of Sciences, Novosibirsk, Russia; Institute of Cytology and Genetics of Siberian Branch of the Russian Academy of Sciences, Novosibirsk, Russia; Institute of Cytology and Genetics of Siberian Branch of the Russian Academy of Sciences, Novosibirsk, Russia; Institute of Cytology and Genetics of Siberian Branch of the Russian Academy of Sciences, Novosibirsk, Russia; Innovation Centre Biruch-NT, Belgorod region, Russia; Institute of Cytology and Genetics of Siberian Branch of the Russian Academy of Sciences, Novosibirsk, Russia

**Keywords:** methylotrophic yeasts, Pichia pastoris, Ogataea, Komagataella, recombinant enzymes, метилотрофные дрожжи, Pichia pastoris, Ogataea, Komagataella, рекомбинантные ферменты

## Abstract

Methylotrophic yeasts have been used as the platform for expression of heterologous proteins since the 1980’s. They are highly productive and allow producing eukaryotic proteins with an acceptable glycosylation level. The first Pichia pastoris-based system for expression of recombinant protein was developed on the basis of the treeexudate- derived strain obtained in the US southwest. Being distributed free of charge for scientific purposes, this system has become popular around the world. As methylotrophic yeasts were classified in accordance with biomolecular markers, strains used for production of recombinant protein were reclassified as Komagataella phaffii. Although patent legislation suggests free access to these yeasts, they have been distributed on a contract basis. Whereas their status for commercial use is undetermined, the search for alternative stains for expression of recombinant protein continues. Strains of other species of methylotrophic yeasts have been adapted, among which the genus Ogataea representatives prevail. Despite the phylogenetic gap between the genus Ogataea and the genus Komagataella representatives, it turned out possible to use classic vectors and promoters for expression of recombinant protein in all cases. There exist expression systems based on other strains of the genus Komagataella as well as the genus Candida. The potential of these microorganisms for genetic engineering is far from exhausted. Both improvement of existing expression systems and development of new ones on the basis of strains obtained from nature are advantageous. Historically, strains obtained on the southwest of the USA were used as expression systems up to 2009. Currently, expression systems based on strains obtained in Thailand are gaining popularity. Since this group of microorganisms is widely represented around the world both in nature and in urban environments, it may reasonably be expected that new expression systems for recombinant proteins based on strains obtained in other regions of the globe will appear.

## Abstract (ru)

Метилотрофные дрожжи используются в качестве платформы для экспрессии гетерологичных бел- ков с 1980-х гг. Они имеют высокий уровень продукции и позволяют получить белки эукариот с приемлемым уровнем гликозилирования. Первая система для экспрессии рекомбинантного белка на основе Pichia pastoris была разработана на основе штамма, выделенного из сокотечения деревьев на юго-западе США. Система распространялась бесплатно в научных целях и применяется во всем мире. В ходе классификации метилотрофных дрожжей по молекулярно-биологическим маркерам штаммы, используемые для получения рекомбинантного белка, были реклассифицированы как Komagataella phaffii. Они находятся в свободном доступе согласно патентному законодательству, однако распространялись на договорной основе. Это делает неопределенным их статус для коммерческого использования и, соответственно, стимулирует поиск альтернативных штаммов для экспрессии рекомбинантного белка. Были адаптированы штаммы других видов метилотрофных дрожжей, среди которых преобладают представители рода Ogataea. Несмотря на филогенетическую удаленность представителей рода Ogataea и Komagataella, во всех случаях оказалось возможным использовать классические векторы и промоторы для экспрессии рекомбинантного белка. Существуют системы экспрессии на основе других штаммов рода Komagataella, а также рода Candida. Потенциал этих микроорганизмов для генной инженерии далеко не исчерпан. Перспективно как усовершенствование имеющихся систем экспрессии, так и создание новых на основе штаммов, выделенных из природных источников. Исторически до 2009 г. в качестве систем экспрессии использовались штаммы, выделенные на юго-западе США. В настоящее время начали развиваться системы экспрессии на основе штаммов, полученных в Таиланде. Поскольку эта группа микроорганизмов широко представлена по всему миру как в природной, так и в городской среде, можно ожидать появления систем экспрессии рекомби- нантных белков, созданных на основе штаммов, выделенных и в других регионах планеты.

## Introduction

Methylotrophs are a group of microorganisms that can use single-carbon methane compounds, such as methanol, methylamine, etc., as the sole source of carbon and energy. The requirement that all C–C bonds are formed enzymatically during cellular metabolism poses a challenge for the cell. Only some microorganisms are capable of doing so, such as Gramnegative proteobacteria and Gram-positive bacteria (Antony, 1986), as well as yeasts (Wegner, Harder, 1987), which employ metabolic pathways for methanol oxidation to produce energy and form C–C bonds. Both in yeasts and bacteria, C–C bonds are formed through the formation of formaldehyde (Yurimoto et al., 2005), a toxic intermediate product that subsequently is either dissimilated into CO_2_ or assimilated into biomass.

Methylotrophic yeasts were discovered in the late 1960s, when methylotrophic bacteria had already been well known. The difference in times of their discovery was mainly caused by the challenges related to yeast isolation and significant bacterial contamination of samples (Trotsenko, Torgonskaya, 2011). The habitats of methylotrophic yeasts are those where the biomass is degraded to give rise to methoxy groups (soil, fallen trees, rotten fruit, etc.). Methanol is produced naturally during methane oxidation (e. g., by methane-oxidizing bacteria in the plant rhizosphere) and pectin or lignin degradation (MacDonald, Fall, 1993; Nakagawa et al., 2000). Most natural isolates of methylotrophic yeasts were detected in tree sap (exudates) or rotting wood (Kurtzman, Robnett, 1998; Kurtzman, 2005).

The interest in methylotrophic organisms for bioengineering applications arose in the early 1970s, when methanol was an inexpensive raw material and was considered a virtually inexhaustible fossil feedstock. However, after the 1973 oil crisis its price never dropped back to the pre-crisis level, and Western countries chose to lower their dependence on hydrocarbons. Plant-based proteins (and soybean protein in particular) came to fore and replaces fodder protein derived from unicellular methylotrophs.

In the 1980s, methylotrophic yeasts became widely used again, although in new areas. Today, they are utilized as a platform for genetic engineering and commercial-scale production of recombinant proteins. Furthermore, methylotrophic yeasts are a convenient object for studying the features of eukaryotic cell organization. Methods for utilizing methylotrophic yeasts in applied areas (as markers of coastal pollution, for treatment of discharge water at sulfate-cellulose and alcohol-and-liquor manufacturing enterprises, for treatment of formaldehydecontaminated air, etc.) are currently being elaborated (Kutty, Philip, 2008; Trotsenko, Torgonskaya, 2012; dos Reis et al., 2018).

Pichia pastoris (Komagataella phaffii) is a methylotrophic yeast species that is most frequently used for scientific research and commercial purposes. These yeasts can consume both sugars and methanol to produce proteins with a high yield. Initially, back in the 1970s, Phillips Petroleum suggested using P. pastoris as a single-cell protein producer due to their ability to form high-density cultures both on glucose and methanol substrates (Mishra, Baranwal, 2009). The maximum cell density achieved during fermentation is higher than 100 g/L (dry basis) (Wegner, 1981). In the 1980s, the P. pastoris-based heterologous expression system was developed with the use of a strong and strictly-regulated alcohol oxidase 1 (AOX1) promoter (Cregg et al., 1985). In combination with the existing fermentation technologies for animal feed protein manufacturing, AOX1 promoter was ensuring an exceptionally high recombinant proteins expression level. An important benefit of using it in recombinant protein manufacturing is the high expression level in the presence of methanol and robust inhibition of the process in the absence of methanol, which makes it possible to regulate the production of target proteins, including the autotoxic ones (Kurtzman, 2009). Production of biomass-derived hydroxynitrile lyase enzyme (the yield being 20 g of recombinant protein per liter of the culture) was one of the first large-scale commercial manufacturing processes established in the 1990s (Hasslacher et al., 1997).

Another advantage of P. pastoris is that it ensures the low glycosylation level. Thus, the S. cerevisae yeast species is no longer used in production of recombinant proteins, since it often hyperglycosylates proteins (up to their complete inactivation) (Darby et al., 2012).

The P. pastoris-based expression system has become widely used in basic research. Among methylotrophic yeasts, the P. pastoris species was the first to be developed. It turned out to be a convenient bioengineering system for production of eukaryotic proteins that are not properly expressed in bacteria. Phillips Petroleum made a forwardlooking decision to make this expression system available to the scientific community for research purposes, which was a major driver for common application of this yeast species as a platform for recombinant protein expression. Histidineauxotrophic strain GS115, reconstituted prototrophic strain X-33, aox1 knockout strains KM71 and KM71H, as well as protease-deficient strains SMD1168 and SMD1168H, and ade2 auxotrophic PichiaPink™ strain, are the most frequently utilized commercially available strains. However, their use for commercial purposes is restricted by material distribution policy. Therefore, searching for alternative options that could be used instead of the licensed P. pastoris strains is quite relevant today.

## Phylogenic analysis

Progress in molecular biology methods as well as mounting evidence on gene sequences for various species made it possible to investigate into phylogeny of methylotrophic yeasts, which was accompanied by a number of surprises.

First of all, gene sequence analysis revealed that yeasts known as Pichia are not monophyletic despite the phenotypic similarity of the majority of species of this genus. First research works discovered that methanol-assimilating species P. pastoris, P. angusta and Hansenula polymorpha are remotely related with one another as well as with Pichia membranifaciens, the typical Pichia species. Yamada et al. (1994, 1995) suggested assigning P. pastoris to a new genus Komagataella and classifying P. angusta as Ogataea polymorpha into a freshly described genus Ogataea. New genera were suggested on the basis of analysis of divergence in partial large subunit (LSU) and small subunit (SSU) ribosomal RNA (rRNA) sequences. Since each analysis was limited to a relatively small number of species, it was unclear how close P. pastoris and P. angusta (H. polymorpha) were related to numerous unstudied species, and therefore suggestions were not accepted (Kurtzman, 1998). However, further analysis of D1/D2 regions in the LSU of the rRNA gene sequences for all now-known ascomycetes confirmed that the Komagataella, Ogataea and Pichia classification is correct (Kurtzman, Robnett, 1998), while phylogenetic divergence demonstrated as a result of single-gene sequence analysis was confirmed during the analysis of multi-gene sequences (Kurtzman et al., 2008). Therefore, Komagataella, Ogataea and Pichia are three separate genera. Thus, Komagataella pastoris is the most correct name from the viewpoint of taxonomy. However, due to historical reasons, P. pastoris remains most wide-spread today.

Secondly, it turned out that the name P. pastoris covers at least two yeast species, i. e. Komagataella phaffii and K. pastoris (Cregg et al., 1993; Kurtzman, 2009). The gene sequence analysis also revealed that these are not the only Komagataella representatives. K. pseudopastoris initially described as P. pseudopastoris (Dlauchy et al., 2003) was also classified as part of Komagataella on the basis of LSU and SSU rRNA sequence analysis. Similarly, K. phaffii was classified as Komagataella on the basis of the analysis of the D1/D2 fragment in the LSU rRNA gene (Kurtzman et al., 2008).

Evolution of parallel sequencing methods helped to build up the database on genome sequences of a great number of microorganisms (including methylotrophic yeasts), which allows for a more detailed phylogenetic analysis. 18S rRNA gene sequence is the most wide-spread and conservative marker for analysis of phylogenetic relations among various fungi representatives. Relevant genes were extracted from genomes of methylotrophic yeasts part of the NCBI database and used for the phylogenetic tree design (see the Figure).

**Fig. 1.**
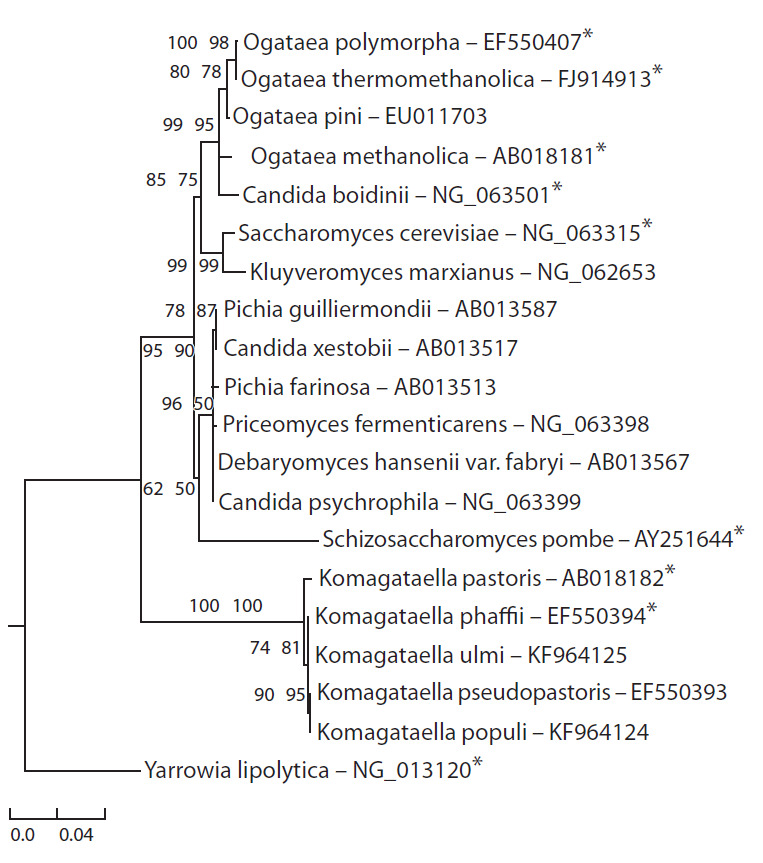
Phylogenetic tree built on the basis of sequences of ribosomal 18S gene of Saccharomycetaceae species with the help of maximum likelihood method. SH-Like aLTR and UfBoot support ratios are expressed in percentage on tree branch joints. Figures to the right of species names are identification numbers for relevant sequences in the NCBI data base.

Nucleotide sequences were evened with the help of MAFFT algorithm in MAFFT v7.312 program (Katoh, Standley, 2013) with –localpair and –maxiterate 1,000 parameters. Phylogenetic tree was designed on the basis of the maximum likelihood method with the help of IQ-TREE program (Trifinopoulos et al., 2016). –Auto and +R (FreeRate heterogeneity) parameters were used to determine the best patterns of nucleotide replacement models, minimum value of the Akaike criterion (AICc) being the key parameter. Two ratios, i. e. SH-like aLRT (1,000 replications) and ultrafast bootstrap (UfBoot, 1,000 replications), were used for statistical support of maximum likelihood in the IQ-TREE program.

As one can see, methylotrophic yeast used for production of recombinant proteins split into three groups: Pichia, Ogataea and Komagataella (see the Figure). Also, one can derive at a conclusion that Ogataea is phylogenetically more heterogeneous in comparison to Pichia and Komagataella.

## Methylotrophic yeasts used for production of recombinant proteins as alternative strains, and their occurrence in nature

P. pastoris (K. рhaffii and K. kurtzmanii ). The P. рastoris yeasts group, the majority of representatives of which have been reclassified as Komagataella representatives, is most well-studied among methylotrophic yeasts. Its typical habitat is tree sap (exudate) in regions from moderate to tropical. Initially, P. pastoris was isolated from chestnut exudates in France. Later, it was discovered to occur widely in Hungary and USA (Spencer et al., 1996; Negruta et al., 2010; Kurtzman, 2011a). Besides, P. pastoris yeasts were discovered in exudates of oil palm in Nigeria (Faparusi, 1974), sap of white algarrobo in Argentina (Spencer et al., 1995), and sap of red oak in Canada (Bowles, Lachance, 2007). The zoning of their occurrence is very curious: this is a predominant species of methylotrophic yeasts in the woods of the Pacific coast of the American Northwest; they are present (although not predominant) in Europe, Africa and South America, and are completely missing in Japan (Lachance et al., 1982).

Although the P. pastoris-based expression system patented by Philips Petroleum is wide-spread, alternative strains of methylotrophic yeasts are being searched for. Those are required for invention of new unlicensed and thus applicable for commercial use platforms for recombinant proteins production.

P. pastoris (K. phaffii) CBS7435 strain is the closest to those previously suggested by Philips Petroleum. Strains derived from it are unlicensed (Ahmad et al., 2014). At the same time, this strain is a predecessor of patented strains most widely used for production of recombinant proteins these days. On the one hand, this paves the way for use of CBS7435 compounds thanks to the vast knowledge base built up with their help, and on the other hand, not all genome modifications for this strain can be patented taking into consideration their description in academic literature and in patents.

Strain K. kurtzmanii Y-727/KPB 2878/Starmer 75-208.2/ CBS 12817/NRRL Y-63667 was patented in the Russian Federation. It was isolated by Prof. Starmer from fir sap in Arizona mountains, USA (Naumov et al., 2013), and is one of the closest relatives of the P. pastoris (K. phaffii) CBS7435 strain.

P. guilliermondii (Meyerozyma guilliermondii ). P. guilliermondii sporogenous species can be isolated from a variety of sources, i. e. plants, lake water, cow rumen, or oilcontaminated soil. Besides, these yeasts were discovered in elm-dwelling insects, in uncontaminated oil and water (Negruta et al., 2010a), in shrimp and other invertebrates, and in low-salinity sea water (Kutty, Philip, 2008). Before, this yeast species was used in gene engineering as a source of genes that were among other things expressed in P. pastoris (Handumrongkul et al., 1998; Zhang et al., 2009), rather than as an expression system.

Since the majority of methylotrophic yeasts have similar methanol-inducible promoter in methanol utilization paths (Hartner, Glieder, 2006), the research group from Malaysia tested the assumption that expression constructs developed on the basis of K. phaffii could be used for expression of recombinant proteins in other methylotrophic yeasts (Oslan et al., 2015). They isolated the strain called Pichia sp. strain SO from a rotten orange; its SSU sequence demonstrated its 100 % similarity to P. guilliermondii. Then, authors discovered that zeocin can be used as a marker for strain SO (Oslan et al., 2012), and conducted work on cloning the recombinant lipase expression construct. The work continued, and in 2017 an article on optimization of expression of T1 lipase isolated earlier from Geobacillus zalihae with use of P. guilliermondii was published. As the result of this work, T1 lipase yielded a 3-fold increase over medium (Abu et al., 2017).

P. (O.) methanolica was suggested by Invitrogen (USA) as a platform for production of recombinant proteins a little later than the same company suggested P. pastoris. Initially, it was isolated from soil sample in Japan in 1974; strains of this species were also isolated in the USA (Sibirny, 1996; Kurtzman, 2011b). In 2008, yeasts of this group (heterogeneous as P. pastoris) were also isolated on the territory of Russia from willow galls created by slug (Glushakova et al., 2010). P. (O). methanolica didn’t become popular as a platform for production of recombinant proteins although there are single messages about its use, e. g., for expression of human glutamic acid decarboxylase (Raymond et al., 1998). Probably, the popularity of P. (O). methanolica as a platform for recombinant proteins is low because Invitrogen has a different, P. pastoris-based platform (K. phaffii).

Hansenula (O.) polymorpha is a thermotolerant methylotrophic yeast able to grow at temperatures below 50 °С wide-spread in nature. Besides such media as rotting fruit and other plants typical for methylotrophic yeasts, one of typical habitats for H. polymorpha is organism of insects, including Drosophila melanogaster typically used in research (Spencer J., Spencer D., 1997). Since H. polymorpha demonstrates good growth at high temperatures, it could possibly be found around hot springs and in tropical areas.

In science, these yeasts have been used as a model organism for studying peroxisome biogenesis and degradation mechanisms, methanol metabolism control, assimilation of nitrates and reaction to stress (van der Klei et al., 2006). H. polymorpha turned out rather effective for production of recombinant proteins as well (Gellissen, 2005). Recombinant antigen of the hepatitis B virus (HBsAg), that was successfully commercialized under HepaVax-Gene and AgB trademarks (Seo et al., 2008), is the most significant therapeutic protein produced with the help of H. polymorpha. Producers of recombinant proteins with high potential for pharmaceutical purposes were developed on the basis of H. polymorpha: hirudin from leech Hirudinaria manillensis (Weydemann et al., 1995) and some human proteins including α1-antitrypsin (Kang et al., 1998), IFNα-2a (Degelmann et al., 2002), serum albumin (Kang et al., 2001), epidermal growth factor (Heo et al., 2002) and parathyroid hormone (Sohn et al., 2012). Besides medical proteins, there were developed producers of food and commercial enzymes: hexose oxidases (Cook, Thygesen, 2003), phytases (Mayer et al., 1999), levansucrase from Zymomonas mobilis (Park et al., 2004), and glucose oxydases from Aspergillus niger (Kim et al., 2004).

P. (O.) thermomethanolica. Besides strains utilized by Invitrogen, there is O. thermomethanolica BCC16875 strain that can be considered one of best-studied methylotrophic yeasts alternatives for production of recombinant proteins. Knowledge of occurrence of O. thermomethanolica is very scarce as this yeast species was discovered only recently, in 2005, in soil samples in Thailand (Limtong et al., 2013).

For the first time, information about strain BCC16875 was published by a research group from Thailand in 2012. The research focuses on testing the possibility of using biomolecular tools for accumulation of protein in this strain. Classical methanol-inducible alcohol oxidase (AOX1) promoters and constitutive glyceraldehyde 3-phosphate dehydrogenase (GAP) promoters utilized for working with P. pastoris were shown to drive efficient gene expression in this new strain. Recombinant phytase and xylanase were expressed from both promoters as secreted proteins, with the former demonstrating different patterns of N-glycosylation dependent on the promoter and culture medium used. The major glycoprotein oligosaccharide species produced from O. thermomethanolica BCC16875 is Man8-12GlcNAc2 that is similar to that of other methylotrophs. Moreover, mannosylphosphate and α-1,6- and α-1,2-linked mannose modifications of heterologous secreted protein were also detected. The level of expression of recombinant protein turned out to be equal to the level of expression of commercial strains, which makes the suggested platform a good alternative to widely used Invitrogen’s strains (Tanapongpipat et al., 2012).

Studies of the suggested strain O. thermomethanolica BCC16875 continued during next following years. Promoters typical for this train were studied (Harnpicharnchai et al., 2014; Promdonkoy et al., 2014), as well as methods of highdensity cultivation for expression of recombinant proteins (Charoenrat et al., 2016). Besides, work on optimization of strain’s metabolism for increase of target products’ output was started. Thus, the level of expression of auxiliary proteins of endoplastic reticulum was increased for this purpose (Roongsawang et al., 2016). Studies of the strain became especially active after 2016, and today there is a significant number of works on updating it to the present-day level as an expression system. In 2018, the CRISPR-Cas9 system was adapted for this strain (Phithakrotchanakoon et al., 2018b), a new sucrose-induction-based expression system was developed (Puseenam et al., 2018; Boonchoo et al., 2019), and studies of metabolism at proteomic and transcriptomic levels continued (Phithakrotchanakoon et al., 2018a).

Candida boidinii is the first described species of methylotrophic yeasts. It is also apparently most wide-spread in natural habitat (Ogata et al., 1969). Mainly it’s various plant substrates (tree sap, rotten fruit, some flowers). These yeasts are also abundantly present in naturally fermented olives (Coton et al., 2006). Methylotrophic yeasts are also found in cacti. Their spoiled parts happen to host both C. boidinii, and O. polymorpha, although these species do not prevail among yeasts discovered in these yeast samples. In addition to regular occurrences of methylotrophic yeasts, С. boidinii is also an important marker of seashore contamination. Their lines are predominant in many water and sand samples in Brazil (Kutty, Philip, 2008).

According to the phylogenetic tree of 18S rRNA gene sequences presented in this review, C. boidinii could be classified as Ogataea (see the Figure). Up to 2009, C. boidinii was developed as a platform for production of recombinant proteins alternative to P. pastoris by a group of Japanese scientists (Yurimoto, Sakai, 2009). C. boidinii-based recombinant protein expression system has some characteristics that can be useful in comparison to other methylotrophic yeasts. Level of expression in C. boidinii varies depending on the source of carbon: AOD1 promoter demonstrates high level of expression in methanol-grown or methanol-glycerolgrown cells, medium level of expression in glycerol-grown cells and zero expression in case glucose- or ethanol-grown cells are used as source of carbon (Sakai et al., 1995; Yurimoto et al., 2000). The level of expression is significantly higher for C. boidinii than for O. polymorpha. Besides, high level of expression can be ensured in C. boidinii in case of methanol + glycerol medium, which allows shortening the time for high-density cell cultivation. In case of P. pastoris, glycerol suppresses expression of methanol-induced genes, and therefore control over complete eating of glycerol in the culture prior to methanol induction is required. A strain with knocked-out vacuolar proteinase A (PEP4) and proteinase B (PRB1) is available for both P. pastoris and C. boidinii (Komeda et al., 2002). During studies with use of C. boidinii genes expression system, there were developed strains for production of toxic proteins, i. e. membrane-bound peroxisome allowing to cumulate toxic proteins (Nishikawa et al., 2000; Yurimoto et al., 2001), as well as effective secretion system for production of active transglutaminase (Yurimoto et al., 2004).

## Obtainment of methylotrophic yeasts from natural sources

Yeasts are isolated from water, seawater, atmosphere and ground habitats. They dwell in rotting vegetables and fruit, in moulds, exudates of trees and their barks, in xylophage insects, pig’s intestine, milk of cows suffering from mastitis, in forest, garden and swampy soils, especially drenched with sewage waters, in sea weed and so on (Negruta et al., 2010; Trotsenko, Torgonskaya, 2011). These environmental preferences are most likely due to discharge of methoxyl groups during degradation of lignin and pectin (Nakagawa et al., 2005). In addition to fruit juices and soil samples, methylotrophic yeasts are also found in food (Mu et al., 2012; Kozhakhmetov et al., 2016; Syromyatnikov et al., 2018).

Many types of yeast are wide-spread while some are limited only to a certain narrow habitat. They rarely occur in nature in absence of micellar fungi and bacteria. Therefore, to obtain them one must use selective methods allowing yeasts to have advantages in growth speed. When media for selective isolation of yeasts are developed, low рН is usually used as in the majority of cases yeasts prevail over bacteria in such conditions. Media could also include antibiotics for suppression of bacteria and fungistatic agents for suppression of moulds (Kurtzman et al., 2011).

When yeasts are present in great amounts, they can be isolated by direct application of the material or its suspension on sour agarized medium that can also be enriched with antibiotics or have other selecting properties. Agar hydrolyzes in low-рН medium during autoclaving. Therefore agar and medium are sterilized separately, cooled to around 45 °С, mixed and distributed among Petrie dishes. The majority of yeast species can be isolated at 3.7 рН, but some species such as Schizosaccharomyces species require higher рН ranging from 4.5 to 5.0. If yeasts are present in the sample in low quantities, their population could be increased by preliminary incubation of the sample in liquid medium at рН up to 3.8 (Kurtzman et al., 2011).

To isolate specific physiological groups of yeasts, it’s necessary to find additional selecting parameters. Methylotrophic yeasts can be selected with use of methanol as the sole source of carbon and energy in the medium. Thus, when methylotrophic yeasts were isolated from grape leaves in Thailand, YNB medium with extra 0.5 % of methanol was used to get the enrichment culture. Cultivation continued for 4–5 days at 27 °С, following which enrichment cultures were spread on 0.5 % v/v methanol-YNB agar. As a result, 2 new species were isolated that classified as Ogataea (Limtong et al., 2013).

## Conclusion

Methylotrophic yeasts are wide-spread as a platform for production of recombinant protein. Initially, they became of interest to biotech companies as single-cell protein producers. However, due to the 1973 oil crisis, methanol grew in price and isolation of feed protein from it became irrelevant. At the same time, due to discovery of proteins key to molecular biology such as thermotolerant polymerases, lygases and restrictases, various microorganisms modification methods started being developed, including those for production of recombinant proteins.

Production of proteins with use of microorganisms for various purposes, in the first place, for food and animal feed as well as technological purposes, has been actively developing since 1940s. In the first place, this was connected with the use of natural producers, upgraded in many cases with the help of undirected mutagenesis methods. As molecular biology methods developed, it became possible to develop protein producers untypical for a specific organism (heterologous or recombinant proteins), including with use of yeasts.

Following this trend, Phillips Petroleum developed their own P. pastoris-based expression system for production of recombinant proteins (later renamed into Komagataella phaffii). Thanks to its outstanding properties as well as Phillips Petroleum’s decision to allow its wide-spread utilization for scientific purposes around the world, it got widely popular as a recombinant protein production platform.

Understanding of key properties that made P. pastorisbased expression systems popular is important for further development of protein-expressing platforms. Number one is high protein production level. It’s lower than that of bacteria and micellar fungi but higher than that of other systems, i. e. cells of mammals, plants and insects. Number two: yeasts are eukaryotes, and they have all cell compartments necessary for synthesis and assembly of eukaryotic proteins, which allows them to synthesize proteins that cannot be synthesized with use of bacterial expression systems. Number three is extracellular protein’s ability to synthesize, which brings the costs of the production process down. Number four is the low level of glycosylation of proteins in comparison to many other types of yeast.

Other methylotrophic yeasts, except K. phaffii, have also been tried out as a platform for expression of recombinant protein. In the first place, it should be noted that P. pastoris strain CBS7435 initial for the Invitrogen’s system was suggested to be used as a patent-independent platform. In Russia, Komagataella kurtzmanii strain of American origin was patented. Alternative Ogataea-based expression platforms were developed in the USA: Hansenula (Ogataea) polymorpha and Pichia (Ogataea) methanolica, the latter belongs to Invitrogen. In Japan, there was developed an alternative platform on the basis of Candida boidinii. This system was being developed until 2009, but since then this organism has not been mentioned as a platform used for production of recombinant protein. In Malaysia, strain P. guilliermondii (Meyerozyma guilliermondii) was suggested for production of protein.

One can notice that all yeast strains used for production of heterologous proteins up to 2009 have American origin. This sets a question, whether or not American isolates have unique properties allowing for production of protein in yeasts. More recent works witness that it’s not accurate. First strains were developed by American researchers working in one state. Actually, rapid development of bioscience, including discovery of new methods of molecular biology, took place in the US Southwest. It seems that this fact was the key reason for using strains of this region specifically. The Russian patent is actually the copy of known systems, which requires utilization of the closest species that in the majority of cases have geographically similar habitats.

Overall, alternative methylotrophic-yeasts-based systems haven’t become popular. Reasons are multiple. Lack of interest on part of recombinant proteins market players and disability of new players to enter this market with a new system along with lack of acute need for it is the main reason. For example, Chinese manufacturers actively use strains obtained on the basis of Invitrogen’s strains. It shall also be noted that modern strains may be significantly genetically modified to ensure higher yields of recombinant proteins.

## Conflict of interest

The authors declare no conflict of interest.
